# Toxicity Profiling Validates Trajectory Modeling for Identifying *Sogatella furcifera* Migration Sources in Southern China

**DOI:** 10.3390/insects16111129

**Published:** 2025-11-04

**Authors:** Jian Zhu, Pengqi Quan, Yan Wu, Chao Li, Mingyong Ma

**Affiliations:** 1 Institute of Plant Protection, Hunan Academy of Agriculture Sciences, Changsha 410125, China; zjnjau@163.com (J.Z.); 13213502940@163.com (P.Q.); 2 Guizhou Key Laboratory of Agricultural Biosecurity, Guiyang University, Guiyang 550005, China; 15150535703@163.com; 3 Institute of Farmland and Agricultural Environment Ecology, Hunan Academy of Agriculture Sciences, Changsha 410125, China; hnchaoli0419@163.com

**Keywords:** *Sogatella furcifera*, insect migration, trajectory simulation, toxicity assays

## Abstract

*Sogatella furcifera* (Horváth) is a major migratory rice pest, yet accurate source attribution for this insect remains a persistent challenge. To assess the accuracy of trajectory modeling in determining the source areas of *S*. *furcifera*, we examined an early-season migration event into HN in May 2024. Backward trajectories were simulated from 15 sites in HN, and insecticide toxicity assays were conducted to determine LC_50_ values for pymetrozine and nitenpyram in 26 field populations collected from HN and GX. Trajectory analyses indicated that northwestern HN populations were traced primarily to western GX and northern Vietnam, central HN populations to central/southern GX, western GD, and northern Vietnam, and southeastern HN populations to northeastern/southeastern GX and western GD. Critically, toxicity tests confirmed the trajectory predictions. In particular, no statistically significant differences in LC_50_ values were detected between HN populations and their identified source areas for either insecticide, while significant differences occurred in comparisons with non-source regions for both insecticides. These findings demonstrate that combining trajectory modeling with toxicity testing effectively identifies *S*. *furcifera* source areas during early migration. This study confirms the utility of trajectory analyses and provides an actionable strategy for monitoring and early warning systems targeting migratory pests.

## Simple Summary

Migratory pests contribute substantially to global crop yield losses, making effective monitoring and early warning systems essential for managing their population densities. Trajectory modeling is widely used to identify the source areas of migratory insects, though its precision can be strengthened by incorporating independent validation data. To address this issue, we augmented traditional trajectory analyses with insecticide toxicity assays. Specifically, we evaluated the toxicity of two classes of insecticides (estimated as LC_50_ values) against field-collected white-backed planthopper (*Sogatella furcifera*) populations (Hunan (HN) and Guangxi (GX) provinces, May 2024). Early-season *S. furcifera* in HN originated primarily from multiple rice-growing regions in GX, with additional sources in northern Vietnam and western Guangdong (GD). Importantly, no statistically significant differences in LC_50_ values for either insecticide were detected between source (GX) and destination (HN) populations. Of note, there was strong consistency between trajectory modeling and toxicity data. Collectively, these findings validate the reliability of the trajectory analysis and suggest that insecticide susceptibility profiles may serve as effective biological tracers for identifying migration source areas.

## 1. Introduction

The increasing severity of agricultural pest outbreaks, driven by global warming and pesticide resistance, has become a major contributor to global crop yield losses [1,2,3]. In China, most economically important pests possess migratory abilities. Eleven insect species have been officially designated as Class I agricultural pests based on damage potential, eight of which are migratory species with substantial impacts [4,5,6,7]. Due to their cryptic behavior, migratory pests often cause sudden large-scale outbreaks. Timely and accurate monitoring and early warning systems are recognized as the most cost-effective and environmentally sustainable control strategy [4]. Nevertheless, effective early warning depends fundamentally on a precise understanding of migration dynamics—including source areas, migratory routes, timing and locations of immigration, and population size [8,9]. However, wider adoption of monitoring systems depends on improving the accuracy of migration source identification, which more reliable analytical methods could enable.

The white-backed planthopper, *Sogatella furcifera*, is a highly destructive migratory species that is widely distributed across Asia, northern Australia, Pacific Islands, and Egypt [10]. *S. furcifera* causes direct feeding damage through sap-sucking, leading to leaf yellowing and “hopper burn,” while also transmitting Southern rice black-streaked dwarf virus [11,12]. These impacts frequently cause regional yield losses. Extensive studies have characterized the migration patterns of *S. furcifera* in East Asia to inform control strategies. A multigenerational migration cycle has been established; populations migrate northward from the Indochina Peninsula to high-latitude regions (e.g., northeastern China, Japan, and Korea) in the spring, propelled by southwesterly airflows northwest of the Western Pacific Subtropical High; reverse southward migration occurs in the autumn under northeasterly winds [13,14,15,16,17,18,19,20]. The long-distance migration capacity (>1000 km) contributes to frequent sudden regional outbreaks. Thus, analyses of localized migration processes, particularly immigration pathways and source areas, are needed to support outbreak management.

Identifying source areas is critical for the coordinated cross-regional control of migratory pests [9]. However, individual tracking techniques are largely applicable to larger insects (e.g., butterflies and dragonflies), making migration studies inherently challenging for most micro-insects [21]. Mark-recapture studies have been used to evaluate *Mythimna separata*, *Agrotis ypsilon*, and *Loxostege sticticalis* [22,23,24]. While still considered the most direct approach for confirming migration routes, its application has been limited since the late 20th century owing to prohibitive labor and resource requirements [9]. Trajectory modeling has therefore become an essential tool for investigating insect migration pathways and outbreak mechanisms [25,26]. Model precision continues to improve through parameter optimization [27] and integration of insect radar data, which reveal otherwise unobservable flight behaviors [28,29]. Additional techniques (e.g., pollen/isotope tracing and molecular markers) have also been applied to source attribution [30,31,32,33,34,35]. Despite providing robust evidence for broad migration routes, these methods generally lack precision in pinpointing source locations and often prove technically complex, limiting widespread implementation.

Chemical control is the primary strategy for managing agricultural pests. However, excessive and inappropriate insecticide use accelerates resistance development, increasing application rates and environmental impacts [36]. Owing to its vast territory, China encompasses diverse climates, geographies, and rice cultivation systems, resulting in significant regional variation in pest occurrence and management practices [37]. Consequently, migratory pests encounter spatially and temporally heterogeneous insecticide selection pressures during migration [38]. Using rice stem dip assays, Li et al. [39] observed high chlorpyrifos resistance (Resistance Ratio range 44.75–304.17) in central Chinese *S. furcifera* populations in 2019–2020. Similarly, Liu et al. [40] documented moderate resistance to thiamethoxam (Resistance Ratio range 0.8–21.1) and chlorpyrifos (Resistance Ratio range 0.1–88.2) across 55 populations in southern, eastern, and southwestern China (2014–2022). These field surveys confirmed substantial spatial and temporal variation in insecticide resistance among *S. furcifera* populations.

Given escalating threats from migratory pests, such as *S. furcifera*, developing more reliable source-tracing methodologies is imperative for monitoring and integrated management. Otuka et al. [41] documented insecticide resistance variation in migrant populations of the brown planthopper (*Nilaparvata lugens*) in Taiwan, linking resistance levels to their geographic origins. HN, situated in the middle Yangtze River basin, serves as a critical spring migration corridor and primary infestation zone for *S. furcifera*. To clarify migration sources and insecticide susceptibility in this region, we applied an integrative approach combining trajectory modeling with toxicity testing in spring 2024. (1) Trajectory modeling was used to reconstruct early-season migration pathways to 15 HN monitoring sites. (2) Based on the trajectory analysis, 26 field populations were collected from immigrant and putative source regions. (3) Toxicity assays established LC_50_ values for pymetrozine and nitenpyram. This work clarifies local population dynamics, supports resistance management, and provides a scientific basis for forecasting outbreaks of *S. furcifera* and other migratory pests.

## 2. Materials and Methods

### 2.1. Data Sources

**Figure 1:**
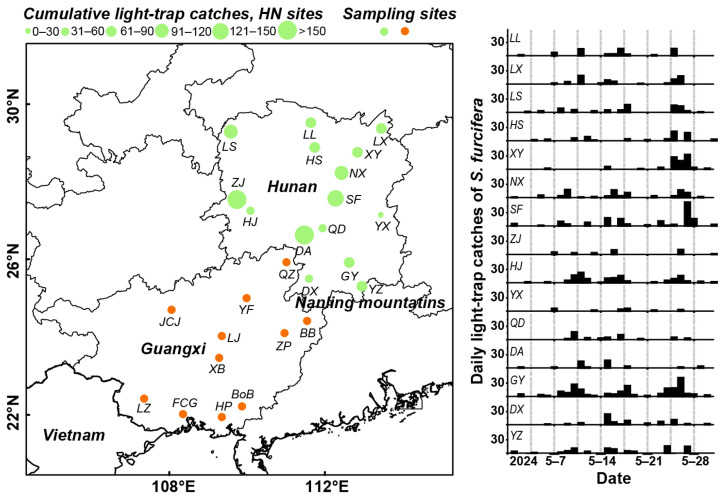
Distribution of monitoring sites in HN and sampling sites. (LX: Linxiang, XY: Xiangyin, NX: Ningxiang, LL: Linli, LS: Longshan, HS: Hanshou, HJ: Hongjiang, QD: Qidong, ZJ: Zhijiang, DA: Dong’an, DX: Daoxian, SF: Shuangfeng, GY: Guiyang, YZ: Yizhang, YX: Youxian, QZ: Quanzhou, YF: Yongfu, LZ: Longzhou, LJ: Liujiang, BB: Babu, ZP: Zhaoping, XB: Xingbin, JCJ: Jinchengjiang, FCG: Fangchenggang, HP: Hepu, BoB: Bobai).

Light trap data for *S. furcifera* in HN during May 2024 were provided by the provincial plant protection monitoring network, which encompassed 15 sites all equipped with 20W blacklight lamps. The monitored locations were as follows (Figure 1): Linxiang County (29.34° N, 113.42° E), Xiangyin County (28.71° N, 112.80° E), Ningxiang County (28.15° N, 112.35° E), Linli County (29.49° N, 111.61° E), Longshan County (29.22° N, 109.50° E), Hanshou County (28.84° N, 111.71° E), Hongjiang County (27.25° N, 110.09° E), Qidong County (26.80° N, 111.95° E), Zhijiang Dong Autonomous County (27.40° N, 109.60° E), Dong’an County (26.49° N, 111.32° E), Daoxian County (25.50° N, 111.59° E), Shuangfeng County (27.46° N, 112.17° E), Guiyang County (25.59° N, 112.59° E), Yizhang County (25.27° N, 112.92° E), and Youxian County (27.17° N, 113.48° E).

Importantly, because early-season pest pressure prior to June is consistently low, insecticide applications targeting *S. furcifera* are exceptionally rare in HN during this period. With early rice transplanting in Hunan taking place in late April, *S. furcifera* populations immigrating before May fail to establish. Consequently, only May light trap records were incorporated into this study. This temporal restriction was deliberately applied to minimize potential confounding from local insecticide exposure on susceptibility profiles—thereby ensuring that resistance levels in monitored populations accurately reflected source-region characteristics rather than post-immigration selection pressures.

### 2.2. Parameters for Trajectory Simulation

The HYSPLIT model (Hybrid Single-Particle Lagrangian Integrated Trajectory Model) was utilized for backward trajectory reconstruction of *S. furcifera*. This model was jointly developed by the NOAA (National Oceanic and Atmospheric Administration) Air Resources Laboratory and the Australian Bureau of Meteorology. This model was originally developed to analyze and calculate the transport and diffusion of atmospheric pollutants. Recently, it has been applied to simulate the migration trajectories of insects, including the oriental armyworm and mosquitoes [5,42]. The migration biological characteristics and trajectory simulation parameters for *S. furcifera* were determined as follows based on established literature. Trajectory simulations were performed on dates corresponding to light trap captures of ≥10 individuals per night.

(1) Flight direction: Due to their small body size, migratory directions of *S. furcifera* are predominantly wind-borne, with individuals transported downwind [15].
(2) Takeoff time: Migration takeoff in rice planthoppers occurs predominantly at crepuscular hours, with peak activity at dusk around 19:00 Beijing Time, corresponding to local sunset [43].
(3) Flight altitude: Aircraft data show rice planthoppers migrate at 300–2500 m altitudes, with concentrations below 1000 m [6,43]. Thus, 1000 m was selected as the representative flight height in this study.
(4) Migration duration and landing time: Rice planthoppers are obligate one-way migrants whose journeys are typically completed within 24 h of takeoff, though landing may occur at any point during this period. Their flight is constrained by a lower temperature threshold of 16.5 °C [6,44], resulting in potential landing times between 20:00 on the departure day and 19:00 the following day.

Meteorological input data were obtained from the NOAA GDAS (Global Data Assimilation System) at 1° resolution (global, 2006–present).

### 2.3. Toxicity Testing

#### 2.3.1. Insect Sources

A total of 26 *S. furcifera* populations were collected from rice paddy fields. Sampling was conducted from 25 to 28 May 2024, corresponding to the period of peak seasonal abundance in HN: (1) 15 HN sites (identical to light trap locations); (2) Guided by trajectory modeling outputs, 11 source regions in Guangxi (GX) were identified and sampled. Among these, nine were located within the distribution area of backward trajectory endpoints: Fangchenggang (22.01° N, 108.35° E), Hepu (21.94° N, 109.34° E), Bobai (22.22° N, 109.87° E), Liujiang (24.02° N, 109.34° E), Xingbin (23.46° N, 109.28° E), Longzhou (22.42° N, 107.35° E), Zhaoping (24.10° N, 111.96° E), Babu District (24.42° N, 111.55° E) and Jinchengjiang District (24.70° N, 108.06° E). For comparative purposes, two additional sampling sites were established outside this distribution area: Yongfu (24.99° N, 109.98° E) and Quanzhou (25.93° N, 111.01° E) (see Figure 1 for the geospatial distribution).

*S. furcifera* adults were sampled using beat sheets from rice stem bases. In Hunan, collections were restricted to areas within 1 km of light traps, whereas Guangxi sampling encompassed entire counties. Each site yielded at least 30 adults. To ensure age uniformity, collected insects (F1) were maintained under controlled insectary conditions (26.0 ± 1.0 °C, 75 ± 5% RH, 16L:8D photoperiod); third-instar nymphs from the resulting F2 generation reared on TN1 (Oryza sativa cv. Taichung Native 1) rice seedlings were then used in bioassays [40].

#### 2.3.2. Insecticides

Insecticides were selected based on: (1) frequent large-scale use in southern Chinese rice regions (particularly GX/HN) and (2) documented resistance development in *S. furcifera* populations. Pymetrozine (95% purity, Anbang Agrochem Co., Huaian, China) and nitenpyram (96.5% purity, Chunguang Agrochem Co., Anyang, China) were used for toxicity testing.

#### 2.3.3. Insecticide Toxicity Assay

The rice stem dip method was performed as follows. (1) Two insecticides were dissolved in acetone with 10% Tween-80 emulsifier, then serially diluted with distilled water to five concentrations. (2) Healthy rice plants at tillering-booting transition were uprooted, root-washed, and sectioned into 10.0 ± 0.5 cm stems. Three stems were grouped and air-dried until surface moisture evaporated. (3) Stems were immersed in different insecticide concentrations for 30 s separately (using distilled water as the control). Treated stems were air-dried for 1 h and transferred to 500 mL plastic cups with moistened cotton sheaths protecting roots. (4) Twenty synchronized 3rd-instar nymphs were aspirated into each cup. Cups were initially oriented horizontally for 30 min to facilitate insect settlement and were then positioned vertically. (5) Four replicates per concentration (80 nymphs total) were maintained in environmental chambers (26.0 ± 1.0 °C, Relative Humidity = 75 ± 5%, and Light:Dark = 16:8). Cotton was kept moist to prevent desiccation. Mortality was assessed after 168 h (pymetrozine) and 96 h (nitenpyram). Tests were discarded if control mortality exceeded 10%.

### 2.4. Identification of Valid Source Areas

Trajectory endpoints (potential source areas) were generated by HYSPLIT Lagrangian simulation. However, not all endpoints represent valid sources. Therefore, three criteria were applied beyond terrestrial location: (1) Trajectory endpoints were required to be within rice cultivation areas. Furthermore, the rice crop had to be at a suitable growth stage (excluding sowing and seedling stages) in May. Consequently, any endpoints located north of 25° N were excluded. (2) Macropterous adults must be present to provide emigrant sources, and (3) overlapping 95% confidence intervals of LC_50_ values for pymetrozine or nitenpyram must exist between light trap sites and trajectory endpoint regions.

### 2.5. Statistical Analysis

Toxicity regression equations, LC_50_ values, and 95% CIs were calculated using DPS v18.10 software. Significance of susceptibility differences was determined by non-overlapping 95% CIs (α = 0.05). Based on HN susceptibility patterns (Table 1 and Table 2), 15 sites were assigned to the following three groups: Group A (Western/Northwestern, including Hongjiang, Linli, Longshan, Hanshou, and Zhijiang), Group B (Central/Northeastern, including Dong’an, Shuangfeng, Qidong, Ningxiang, Xiangyin, and Linxiang), and Group C (Southern/Southeastern, including Daoxian, Youxian, Guiyang, and Yizhang).

Valid trajectory endpoints were identified per group using Section 2.4 criteria. Endpoints were imported into ArcGIS v10.8, where point densities per 0.5° × 0.5° grid were quantified using the Fishnet tool. Probability distributions were then interpolated via natural neighbor method [45]. The maps used in this study were obtained from the Standard Map Service provided by the National Geomatics Center of China (https://www.ngcc.cn/).

## 3. Results

### 3.1. Early-Season Population Dynamics of S. furcifera in HN Light Traps

Light trap captures revealed multiple low-abundance migration events of *S. furcifera* across HN in May 2024 (Figure 1). Although daily captures were recorded, the cumulative total across 15 monitoring sites reached only 1345 individuals. Notably, approximately 35.61% of captures occurred within 24–27 May. A total of 51 days with elevated activity (≥10 individuals) were recorded collectively across all monitoring sites. The highest cumulative captures were recorded at Dong’an (230 individuals) and Zhijiang (156 individuals) in southwestern HN, followed by Shuangfeng (141) and Ningxiang (104) in central regions. Significantly lower captures were documented in eastern and northern sites. These spatial patterns suggest predominant southwestern immigration sources in May.

### 3.2. Backward Trajectory Simulation

**Figure 2:**
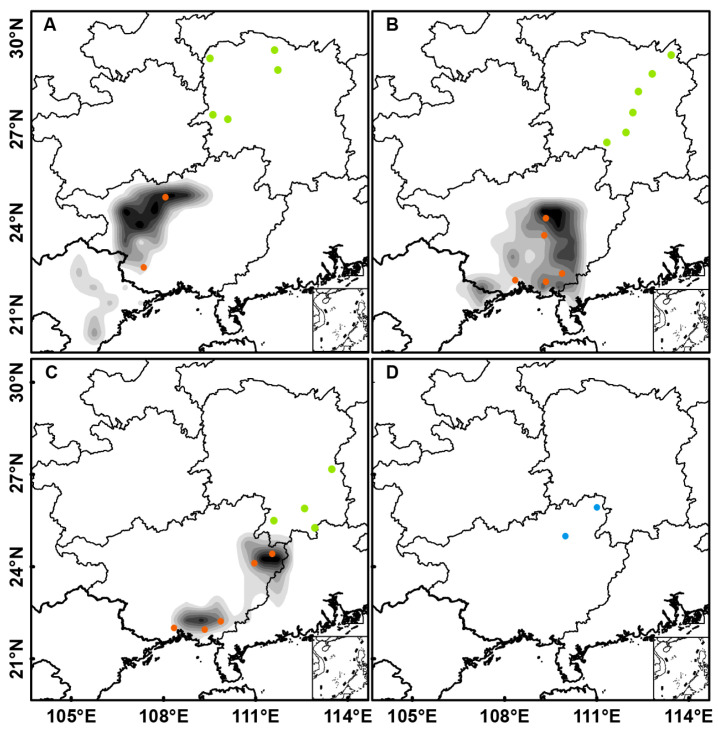
Probability Distribution of Backward Trajectory Endpoints for *S. furcifera* in HN, May 2024 (Dark shading indicates the spatial probability distribution of backward trajectory endpoints, with darker colors representing higher endpoint densities). Starting points of trajectories (green dots): (**A**) Hongjiang, Linli, Longshan, Hanshou, Zhijiang; (**B**) Dong’an, Shuangfeng, Qidong, Ningxiang, Xiangyin, Linxiang; (**C**) Daoxian, Youxian, Guiyang, Yizhang. (**D**) GX field sampling sites located outside the endpoint distribution area (Orange and Blue dots represent sampling sites in GX: orange indicates sites located within the trajectory endpoint distribution area, while blue denotes two additional sites established outside those zones).

Backward trajectory simulations were performed for periods of elevated *S. furcifera* activity at 15 monitoring sites in HN in May 2024. GX, western GD, and northern Vietnam were identified as the primary landing region for the backward trajectories (Figure 2). Specifically, trajectory endpoints from western and northwestern HN (Hongjiang, Linli, Longshan, Hanshou, and Zhijiang) were located primarily in the western rice-growing regions of GX and northern Vietnam, with concentrated clusters in Hechi and Baise (Figure 2A). In contrast, endpoints from central and northeastern HN (Dong’an, Shuangfeng, Qidong, Ningxiang, Xiangyin, and Linxiang) were positioned in the northern Vietnam, western GD, central and southern rice-growing areas of GX, including Laibin, Guigang and Yulin (Figure 2B). Similarly, trajectory endpoints from southern and southeastern HN (Daoxian, Youxian, Guiyang, and Yizhang) were concentrated predominantly in the northeastern and southeastern rice-growing regions of GX and western GD, with core areas identified around Hezhou, Wuzhou and Qinzhou (Figure 2C).

### 3.3. Toxicity Tests

Toxicity tests revealed consistent regional variation in LC_50_ values for pymetrozine and nitenpyram across HN populations (Table 1 and Table 2). Based on susceptibility differences, sites were categorized into three populations (Populations A–C). The 95% confidence intervals for toxicity to pymetrozine and nitenpyram were 24.437–35.801 mg/L and 0.729–1.181 mg/L, respectively, for Population A (Hongjiang, Linli, Longshan, Hanshou, and Zhijiang), 36.703–48.100 mg/L and 1.948–3.056 mg/L for Population B (Dong’an, Shuangfeng, Qidong, Ningxiang, Xiangyin, and Linxiang), and 51.502–58.522 mg/L and 3.236–4.613 mg/L for Population C (Daoxian, Youxian, Guiyang, and Yizhang).

To validate trajectory-derived source areas, 11 GX populations were sampled. Statistical analyses (based on 95% confidence intervals) indicated that there were no significant differences in susceptibility to either insecticide alone between Population A and Longzhou and Jinchengjiang populations (*p* < 0.05), while there were significant differences between Population A and other GX populations (Table 1 and Table 2). Population B did not show significant differences in susceptibility with those of Liujiang, Xingbin, Fangchenggang, Hepu, and Bobai populations. Population C did not exhibit significant differences with Zhaoping, Babu, Fangchenggang, Hepu, and Bobai populations. All HN populations differed significantly from Quanzhou and Yongfu populations in susceptibility.

The trajectory analysis results were strongly validated by the toxicity assay data. Collectively, these findings identified the major source areas for *S. furcifera* populations in HN during May 2024. Specifically, Population A in HN was traced primarily to Longzhou and Jinchengjiang; Population B originated mainly from Liujiang, Xingbin, Fangchenggang, Hepu, and Bobai; and Population C was linked predominantly to Zhaoping, Babu, Fangchenggang, Hepu, and Bobai.

## 4. Discussion

This study demonstrates that early-season *S. furcifera* populations in HN in 2024 predominantly originated from rice-growing regions in western, central, southern, and northeastern GX. Spatial patterns in insecticide susceptibility closely mirrored geographical origins. In particular, Population A (western/northwestern HN) exhibited toxicological profiles similar to those for Longzhou and Jinchengjiang in GX; Population B (central/northeastern HN) showed no significant difference from populations in Liujiang, Xingbin, Fangchenggang, Hepu, and Bobai; and Population C (southern/southeastern HN) aligned closely with populations from Fangchenggang, Hepu, and Bobai, Zhaoping and Babu. The high congruence between trajectory modeling and toxicity test results not only confirms the reliability of the trajectory analysis but also establishes insecticide susceptibility data as a practical indicator for determining source regions of migratory pests. This congruence between trajectory-derived endpoints and toxicological data improves the credibility of source identification—particularly in complex migration systems with overlapping pathways.

Light trap data revealed higher levels of immigrant activity and population densities in southwestern/western HN than in eastern regions (Figure 1). This spatial pattern may be attributed to prevailing southwesterly airflows and the blocking effect of the Nanling Mountains, which likely limited eastern immigration [46]. The findings from our trajectory simulations further validate earlier research findings [16,46], who identified GX as a major source region for early-season *S. furcifera* in HN. Furthermore, our results indicate that a portion of the early-season *S*. *furcifera* population in Hunan Province in 2024 originated from northern Vietnam and western GD (Figure 2). This diversity in source regions complicates accurate source identification and hinders the implementation of cross-regional coordinated control strategies.

Compared with traditional methods, such as mark–recapture studies, pollen/isotope tracing, or molecular genetic markers, trajectory modeling offers significant operational advantages in accessibility and efficiency, substantially reducing labor and resource requirements [9]. Furthermore, Chapman et al. [47] incorporated insect radar-derived flight parameters—including altitude, speed, and orientation—into a trajectory analysis, significantly improving the interpretation of long-distance migration strategies. Zhu et al. [5] demonstrated high consistency between trajectory-simulated autumn return rates of *M. separata* in eastern China and actual light trap capture data, with modeled migration success rates closely aligning with field observations. Despite extensive validation, the accuracy of insect trajectory simulation is not without its constraints, primarily due to two factors: (1) insufficient integration of species-specific biological factors in parameterization and (2) absence of direct ground-truth evidence. In this context, our integration of field-derived LC_50_ data for natural populations with a trajectory analysis provides empirical validation, substantially strengthening the credibility of migration source attribution. Although the LC_50_ values showed only minor variations among the 26 field populations, the statistically significant differences indicate distinct regional origins. Importantly, this conclusion is supported by trajectory modeling results, which align with findings from Otuka et al. [41] in their study on *N. lugens*.

Several methodological limitations must be noted regarding the toxicity assays. First, provided that no insecticide control has been implemented in the immigration areas, sampling should be conducted in both these areas and potential source regions (emigration areas) to avoid distorted resistance profiles. Second, samples were collected immediately following major immigration events to ensure representative susceptibility data. The rice stem dip bioassay is more time-intensive than trajectory analyses, underscoring the need for higher-throughput approaches. Newly developed rapid resistance diagnostic kits [48] offer promising alternatives; however, their accuracy and scalability under operational conditions require further evaluation.

From a broader ecological perspective, migratory insects are subjected to heterogeneous selection pressures across regions, yet gene flow mediated by migration may mitigate resistance development by diluting resistant alleles. Wang et al. [49] reported higher resistance levels in northward-migrating *Plutella xylostella* populations than in southward returning populations, while Yang et al. [38] observed that migration delayed resistance fixation in *M. separata*. Similarly, the similarity in toxicological profiles between HN and GX populations observed herein suggests that migration facilitates gene flow, potentially slowing local resistance evolution through dilution. Conversely, this connectivity also implies that resistance traits arising in one region could spread rapidly through migratory populations, emphasizing the necessity for coordinated regional management strategies. It is therefore recommended that a cross-provincial and international monitoring network be established for *S. furcifera*, integrating trajectory modeling, toxicity testing, and molecular markers to develop a predictive and adaptive management system.

Despite the increasing severity of insecticide resistance and the established role of toxicity assays in evidence-based pest management [39,40], current studies remain limited by short durations and restricted geographical coverage. There is an urgent need for nationally coordinated, sustainably funded resistance monitoring programs. The integration of trajectory analyses and toxicological data can also refine key behavioral parameters, such as takeoff/landing times and migration duration, for other migratory pests. Ultimately, the growing imperative for effective early warning systems for migratory pests should accelerate the development of systematic, large-scale resistance monitoring regimes.

## 5. Conclusions

The accuracy of insect trajectory modeling for determining the source areas of *S. furcifera* was strongly validated using insecticide toxicity assays. Furthermore, susceptibility profiles were identified as a reliable indicator for identifying effective source regions. These findings may promote the implementation of long-term and large-scale insecticide resistance monitoring programs. Collectively, the results precisely identify the geographic origins of *S. furcifera* populations in HN and provide a highly credible and practical framework for tracing the sources of other migratory insect pests. The combined approach exhibits significant potential for broader application in early warning and monitoring systems.

## Figures and Tables

**Table 1:** Toxicity Tests of Pymetrozine to *S. furcifera*.

Site	LC_50_ (95%CI *) (mg/L)	Slope ± SE	*χ* ^2^
Hongjiang (HN)	29.208 (26.377–32.467) a ^†^	3.090 ± 0.304	0.388
Linli (HN)	28.659 (26.026–31.459) a	3.462 ± 0.318	0.913
Longshan (HN)	32.067 (28.960–35.801) a	3.092 ± 0.306	0.067
Hanshou (HN)	27.042 (24.511–29.677) a	3.471 ± 0.322	1.554
Zhijiang (HN)	27.009 (24.437–29.685) a	3.401 ± 0.319	0.587
Dongan (HN)	43.545 (39.446–48.026) b	3.386 ± 0.313	4.131
Shuangfeng (HN)	41.903 (37.682–46.424) b	3.073 ± 0.302	0.588
Ningxiang (HN)	43.690 (39.669–48.100) b	3.404 ± 0.314	2.999
Linxiang (HN)	42.511 (38.605–46.741) b	3.701 ± 0.326	2.887
Xiangyin (HN)	40.737 (36.703–45.029) b	3.157 ± 0.306	0.937
Qidong (HN)	41.588 (37.071–46.449) b	2.805 ± 0.293	0.352
Daoxian (HN)	55.945 (53.516–58.417) c	7.407 ± 0.696	1.071
Youxian (HN)	56.131 (53.789–58.522) c	7.764 ± 0.711	0.945
Guiyang (HN)	54.250 (52.106–56.389) c	8.567 ± 0.755	2.569
Yizhang (HN)	53.910 (51.502–56.283) c	7.427 ± 0.698	0.218
Quanzhou (GX)	18.512 (16.894–20.305) d	3.572 ± 0.322	0.934
Yongfu (GX)	21.341 (19.513–23.373) d	3.667 ± 0.325	1.273
Longzhou (GX)	28.091 (25.568–30.751) a	3.600 ± 0.323	1.74
Jinchengjiang (GX)	31.235 (28.777–33.907) a	4.167 ± 0.351	1.003
Liujiang (GX)	42.548 (38.525–46.867) b	3.308 ± 0.31	1.041
Xingbin (GX)	43.401 (39.246–47.924) b	3.221 ± 0.308	0.098
Fangchenggang (GX)	47.518 (41.579–54.253) bc	2.470 ± 0.221	1.194
Hepu (GX)	47.063 (40.847–54.143) bc	2.297 ± 0.214	0.389
Bobai (GX)	46.959 (40.446–54.429) bc	2.331 ± 0.216	0.224
Zhaoping (GX)	58.090 (55.517–60.802) c	7.051 ± 0.676	1.325
Babu (GX)	57.292 (54.863–59.817) c	7.502 ± 0.697	0.772

Notes: * CI means confidence interval; ^†^ Data in the same row followed by different lowercase were significantly different (*p* < 0.05) by non-overlapping 95% confidence interval.

**Table 2:** Toxicity Tests of nitenpyram to *S. furcifera*.

Site	LC_50_ (95%CI) (mg/L)	Slope ± SE	*χ* ^2^
Hongjiang (HN)	0.906 (0.729–1.096) a ^‡^	3.09 ± 0.304	0.388
Linli (HN)	1.002 (0.922–1.087) a	3.462 ± 0.318	0.913
Longshan (HN)	0.922 (0.851–0.996) a	3.092 ± 0.306	0.067
Hanshou (HN)	0.975 (0.887–1.067) a	3.471 ± 0.322	1.554
Zhijiang (HN)	1.083 (0.991–1.181) a	3.401 ± 0.319	0.587
Dongan (HN)	2.622 (2.379–2.886) b	3.386 ± 0.313	4.131
Shuangfeng (HN)	2.614 (2.389–2.858) b	3.073 ± 0.302	0.588
Ningxiang (HN)	2.815 (2.598–3.056) b	3.404 ± 0.314	2.999
Linxiang (HN)	2.602 (2.393–2.828) b	3.701 ± 0.326	2.887
Xiangyin (HN)	2.767 (2.546–3.007) b	3.157 ± 0.306	0.937
Qidong (HN)	2.392 (1.948–2.897) b	2.805 ± 0.293	0.352
Daoxian (HN)	3.899 (3.566–4.249) c	7.407 ± 0.696	1.071
Youxian (HN)	3.536 (3.236–3.842) c	7.764 ± 0.711	0.945
Guiyang (HN)	4.209 (3.834–4.613) c	8.567 ± 0.755	2.569
Yizhang (HN)	4.071 (3.721–4.443) c	7.427 ± 0.698	0.218
Quanzhou (GX)	0.648 (0.591–0.709) d	3.572 ± 0.322	0.934
Yongfu (GX)	0.649 (0.597–0.705) d	3.667 ± 0.325	1.273
Longzhou (GX)	1.100 (0.983–1.218) a	3.600 ± 0.323	1.740
Jinchengjiang (GX)	1.105 (0.988–1.225) a	4.167 ± 0.351	1.003
Liujiang (GX)	2.623 (2.382–2.872) b	3.308 ± 0.310	1.041
Xingbin (GX)	2.853 (2.318–3.476) b	3.221 ± 0.308	0.098
Fangchenggang (GX)	3.297 (2.679–4.061) bc	2.470 ± 0.221	1.194
Hepu (GX)	3.316 (2.804–3.909) bc	2.297 ± 0.214	0.389
Bobai (GX)	3.260 (2.751–3.847) bc	2.331 ± 0.216	0.224
Zhaoping (GX)	4.140 (3.778–4.526) c	7.051 ± 0.676	1.325
Babu (GX)	3.726 (3.376–4.086) c	7.502 ± 0.697	0.772

Notes: ^‡^ Data in the same row followed by different lowercase were significantly different (*p* < 0.05) by non-overlapping 95% confidence interval.

## Data Availability

The original contributions presented in this study are included in the article. Further inquiries can be directed to the corresponding author.

## Abbreviations

The following abbreviations are used in this manuscript:

LC_50_	Lethal Concentration 50
HN	Hunan province
GX	Guangxi province
GD	Guangdong province
HYSPLIT	Hybrid Single-Particle Lagrangian Integrated Trajectory
NOAA	National Oceanic and Atmospheric Administration
GDAS	Global Data Assimilation System
